# Scar Assessment Tools: How Do They Compare?

**DOI:** 10.3389/fsurg.2021.643098

**Published:** 2021-06-23

**Authors:** Amanda Min Hui Choo, Yee Siang Ong, Fadi Issa

**Affiliations:** ^1^Ministry of Health, Singapore, Singapore; ^2^Department of Plastics, Reconstructive and Aesthetic Surgery, Singapore General Hospital, Singapore, Singapore; ^3^Department of Plastic Surgery and Burns, Buckinghamshire Healthcare NHS Trust, Aylesbury, United Kingdom; ^4^Medical Sciences Division, Nuffield Department of Surgical Sciences, University of Oxford, Oxford, United Kingdom

**Keywords:** clinician reported outcomes, surgical scar, burn scar assessment, scar assessment, patient reported outcome, linear scars

## Abstract

Healing after dermal injury is a complex but imperfect process that results in a wide range of visible scars. The degree of disfigurement is not the sole determinant of a scar's effect on patient well-being, with a number of other factors being critical to outcome. These include cosmetic appearance, symptoms such as itch and pain, functional loss, psychological or social problems, and quality of life. An accurate assessment of these domains can help clinicians measure outcomes, develop, and evaluate treatment strategies. A PubMed literature search was performed up to 31st March 2020. Ten objective scar measurements, four Clinician-Reported Outcome Measures (CROMs), six Patient-Reported Outcome Measures (PROMs), and one combined measure were evaluated for their reliability, clinical relevance, responsiveness to clinical change, and feasibility. Many quantitative tools were limited in their clinical relevance and feasibility, whereas few qualitative CROMs and PROMs have undergone rigorous assessment. This review examines currently available assessment tools, focusing primarily on subjective scar measurements (CROMs, PROMs), and offers a perspective on future directions in the field.

## Introduction

### What Is a Scar?

A scar is the macroscopic disturbance of the normal structure and function of skin, formed following the maturation phase of wound healing (1, 2). An immature scar may initially be pink, hard, raised, and itchy, before maturing over 1–2 years into a paler, softer, flatter, and less itchy lesion. It can be described as atrophic, hypertrophic, and keloid. Linear scars form the largest category of surgical scars, while burn scars are often the most cosmetically and functionally problematic, often due to their traumatic nature, inconsistent pattern, and area (3).

### Why Assess Scars?

A patient's overall satisfaction with wound healing is significantly influenced by the resulting scar. Not only are scars the apparent cosmetic outcome of surgery or injury, but they can also contribute to functional problems. These include movement restrictions that can affect even basic tasks of breathing, eating and speaking. Less visible but important issues include pruritus, pain, and psychosocial sequelae. A scar assessment tool allows the surgeon to consolidate these outcomes. Standardised scales are useful to monitor changes in scar quality over time and to compare scars and the outcome of treatment. The evidence base for scar therapy is limited by the infrequent use of standardised assessment tools across different scar therapies (4). Hence, a holistic and valid assessment tool is essential to ensure progress in wound healing and scar treatment.

## Creating Scar Assessment Tools

Assessment tools must apply a rigorous approach involving patient and clinician input, a pre-test evaluation, modification, and finally application to a target population. A useful assessment tool should be:

- Clinically relevant: It should include items that are important to patients, clinicians, and researchers (*content validity*)
- Reliable: For the tool to be used in large and multiple studies, the same scar should obtain the same results between different raters (*inter-rater reliability*) or subsequent evaluations by the same rater (*intra-rater reliability*). Quantitatively this can be expressed as the Intraclass Correlation Coefficient (ICC) or Kappa coefficient (κ)
- Responsive: If clinicians are to use the tool to assess patients' scars over time or after treatment, the tool should be able to detect these changes. This can be represented by the T-statistic, effect size (ES), Guyatt's responsiveness statistic (GRS), or standardised response mean (SRM)
- Feasible: The target audience (including patients, clinicians, and researchers) should be able to use the tool efficiently and cost-effectively, and easily interpret the results.

## Methods

A literature search was performed in PubMed up to 31st March 2020. The search terms included “(scar assessment tools),” “(scar) and (patient-reported outcome measure),” “(scar) and (clinician-reported outcome measure).” Original studies, literature reviews, and systematic reviews written in English (quantitative and qualitative) were included. Tools that assess surgical, traumatic, and burn scars were identified. The tools could be objective measurements, Clinician-Reported Outcome Measures (CROMs), Patient-Reported Outcome Measures (PROMs), or combined measures that include both clinician- and patient-reported outcome measures. Articles that evaluated effectiveness of scar management therapies were not included. A total of 426 articles were identified, and among these, 21 tools were included: 10 objective scar measurements, four CROMs, six PROMs, and one combined measure. The tools were then evaluated for their reliability, clinical relevance, responsiveness to clinical change, and feasibility (as reported in the literature).

## Current Clinical Assessment Tools

### Objective Scar Measurements

**Scar measurements** most frequently studied in clinical trials (5) are colour (vascularisation and pigmentation), thickness (height: clinical and histological), relief (surface irregularities), pliability (tissue elasticity), and surface area (scar contraction or expansion). The reliability, validity, and responsiveness of these tools are summarised in Table 1A. While these measurements might be seen as an objective and quantifiable way to assess scars, none of the available tools combine clinical relevance and feasibility. Further details of these objective scar measurement tools available are found in Supplementary Materials.

**Table 1A:** Reliability, validity, and responsiveness of existing assessment tools, as reported in the literature reviewed.

	**Reliability**	**Validity**
**Objective measures**		
Colour: Tristimulus reflectance colorimetry (e.g., Minolta Chromameter CR-200/CR-300)	Moderate to good (ICC = 0.73–0.97)	Moderate correlation with POSAS Weak correlation with VSS
Colour: Narrow-band spectrophotometry (e.g., Mexameter)	Good (ICC = 0.95–0.98)	Moderate correlation with VSS
Thickness: Biopsy	Evidence not found	Evidence not found
Thickness: Tissue ultrasound palpation system	Good (ICC = 0.89–0.91)	Moderate correlation with VSS
Relief: Silflo silicon polymer	Evidence not found	Evidence not found
Pliability: Cutometer skin elasticity metre	Poor to moderate (ICC = 0.35–0.76)	Weak to moderate correlation with POSAS
Pliability: Tonometry	Good (ICC = 0.95)	Moderate correlation with VSS
Surface area: scar-tracing on paper	Moderate to good (ICC = 0.48–0.88)	Correlates with changes in healing
Surface area: photography	Moderate to good (ICC = 0.72–0.93)	Correlates with true surface area
Surface area: stereophotogrammetry	Moderate to good (ICC = 0.72–0.93)	Correlates with physical measurements
	Responsiveness not reported for most objective measure	
**Clinician-reported outcome measures**		
Rating colour-side photographs	Good (ICC = 0.94)	Evidence not found
Wound Evaluation Scale (WES)	Moderate (κ =0.31–0.66)	Evidence not found
Manchester Scar Scale (MSS)	Evidence not found	Correlates with histologic scores
Vancouver Scar Scale (VSS)	Moderate (κ = 0.40–0.56)	Validity uncertain
Patient and Observer Scar Assessment Scale (POSAS) (observer-reported domain)	Moderate to good (ICC = 0.73–0.92)	Some evidence of validity
	Only POSAS has preliminary evidence for responsiveness	
**Patient-reported outcome measures**		
POSAS (patient-reported domain)	Moderate to good (ICC = 0.65–0.81)	Did not include patient input in content development
Brisbane Burn Scar Impact Profile (BBSIP)	Moderate to good (ICC = 0.65–0.83)	Preliminary
Burn-Specific Health Scale (BSHS)	Evidence not found	Clinically relevant
Bock Questionnaire	Good (*r* = 0.94–0.96)	Did not include patient input in content development
Patient Scar Assessment Questionnaire (PSAQ)	Moderate to good (ICC = 0.48–0.87)	Conducted patient interviews
Patient-Reported Impact of Scars Measure (PRISM)	Good (ICC = 0.83–0.89)	Utilised qualitative interview data for content construction Lacks appearance domain
SCAR-Q	Preliminary (ICC = 0.88–0.94)	Utilised qualitative datasets, cognitive interviews and expert opinion
	Responsiveness not reported for most Patient-Reported Outcome Measures	

*ICC, Intraclass Correlation Coefficient; κ, Kappa coefficient*.

## Clinician-Reported Outcome Measures (CROMs)

One of the first scar assessment scales was created in 1988 in a burns institute in Boston to measure burn-induced cosmetic disfigurement (6). Colour-slide photographs of 30 burn patients were shown to 95 clinical and non-clinical observers who rated scar irregularity, thickness, discolouration, and overall cosmetic disfigurement.

- Reliability: The study demonstrated that a panel of four to eight observers with at least some experience with burn patients could produce reliable average ratings of burn scars (6).
- Feasibility: The requirement of at least four observers is not feasible in a clinical setting.

Subsequently, a Wound Evaluation Scale was developed to assess repaired lacerations (7). This was a six-item, dichotomous categorical scale.

- Clinical relevance: Unfortunately, the assessed variables reflect surgical attributes rather than scar appearance, and have less relevance to patients or clinicians.

Eventually, more sophisticated scales emerged. The **Manchester Scar Scale** (MSS) is a multi-item categorical scale, with a global scar assessment made with a visual analogue scale (VAS) (8). This scale includes descriptors of greater clinical significance, such as contour (flush, indented, hypertrophic, or keloid) as opposed to physical measurements.

- Reliability: The authors' evaluation with 69 patients showed reasonable validity and inter-rater and intra-rater reliability, but this has not yet been demonstrated in further studies.

The Vancouver Scar Scale (VSS) (9) has four variables (vascularity, height or thickness, pliability, and pigmentation) forming a numeric score from 0 to 14.

- Clinical relevance: While it is now one of more commonly-used assessment tools, it is also the most frequently-modified (10), making it difficult to assess its validity.
- Reliability: It has been shown to have acceptable internal consistency and moderate inter-rater reliability.

## Tools Incorporating Patient-Reported Outcome Measures

Despite various improvements, the abovementioned scar assessment tools do not include the patient's experience and assessment. Arguably, patient function, and satisfaction are the most important clinical outcomes.

Given the importance of the patient's perspective, guidelines have now been created for the development and validation of Patient-Reported Outcome Measures (PROMs). The COnsensus-based Standards for the selection of health Measurement Instruments (COSMIN) checklist (11, 12) includes requirements to measure properties such as clinical relevance (content validity), reliability, and responsiveness, as well as standard design requirements and preferred statistical methods.

The Patient and Observer Scar Assessment Scale (POSAS) was developed with the support of the Dutch Burn Foundation (13). The POSAS comprises of two numerical scales: the patient scores scar colour, pliability, thickness, relief, itching and pain; the observer scores scar vascularisation, pigmentation, pliability, thickness, and relief. Both scales also include a general rating of appearance.

- Clinical relevance: Using POSAS at 3 months enabled a prediction of final burn scar quality in one study (14), which can be of clinical utility.
- Reliability: The POSAS showed better internal consistency, inter-rater, and intra-rater reliability as compared to the VSS. The POSAS was then applied to linear scars and showed good internal consistency, reliability, and observer-patient agreement. When compared to the VSS in the assessment of keloid scars, the POSAS showed strong inter-rater reliability and convergent validity (15).
- Feasibility: The POSAS has also been shown to be transferrable to other cultural contexts in a few studies (16, 17).

When POSAS was first applied, an interesting divergence in the patient's and observer's general ratings was found. The total scores from patients were poorer than those from observers. The observers' opinion was significantly influenced by relief, thickness, pigmentation and colour. Conversely, scar thickness and itching significantly influenced the patients' opinion. Thickness and itching were also identified as significant factors for patients with linear scars. Pain and itching are subjective and imperceptible to the observer. There could be more factors relevant to the patient that remain invisible to the clinician and absent in scar assessment tools. Shortly after, POSAS 2.0 was created (with surface area as an additional parameter in the observer scale).

- Reliability: When tested on linear scars, this was found to have stronger reliability, and revealed similar findings to the first study (3).
- Feasibility: It is now the most frequently used CROM and PROM amongst studies of patients with burn, surgical, keloid, and necrotising fasciitis scars (10).

The subjective opinion of the patient could also be influenced by the wider context of the scar. A study on burn patients showed that patients with deeper burns had higher psychologic functioning than patients with superficial burns (18). One way to quantify holistic health is through a Health-Related Quality of Life (HRQoL) measure. The **Brisbane Burn Scar Impact Profile** (BBSIP) was the first HRQoL measure for paediatric and adult burn patients.

- Content validity: It was created through a relatively rigorous process of semi-structured interviews, content validation surveys, and cognitive interviews (19).
- Reliability: The proxy-report measure for patients aged zero to 8 years (BBSIP^0−8^) has been shown to have good longitudinal validity at baseline, 1–2 weeks and 1-month intervals (20).

The Burn-Specific Health Scale (BSHS) is a 114-item scale that quantifies dysfunction and distress across six major health domains in patients following burn injuries.

- Clinical relevance: The BSHS has been widely used and adapted across cultural contexts, suggesting clinical utility in various contexts.
- Reliability: Inter- or intra-rater reliability has not yet been demonstrated beyond initial studies (21).
- Feasibility: While comprehensive, the 114 items covered may make this scale a less feasible tool for large-scale studies conducted in outpatient settings.

Bock is a 15-item questionnaire for paediatric and adult patients with hypertrophic and keloid scars (22).

- Clinical relevance: It did not include patient input in content development, which may reduce its content validity for patients.
- Reliability: Bock has been shown to have good inter-rater reliability (23).

The Patient Scar Assessment Questionnaire (PSAQ) is a 39-item, multi-scale questionnaire for patients with linear surgical scars (24).

- Clinical relevance: PSAQ was created following qualitative patient interviews, showing some assimilation of patient input.
- Reliability: PSAQ has been shown to provide good inter-rater reliability (23).

Patient-Reported Impact of Scars Measure (PRISM) is a 37-item instrument measuring HRQoL and physical symptoms in various types of scars (25).

- Clinical relevance: PRISM demonstrated a rigorous content design and validation process by using qualitative patient data. Additionally, PRISM attempts to encompass both symptomatic and psychosocial aspects of scars.
- Reliability: PRISM has shown good intra- and inter-rater reliability (23).

Most recently, SCAR-Q has been created for children and adults with surgical, traumatic, and burn scars (26). The researchers utilised qualitative datasets to identify three key domains (scar appearance, scar symptoms, and psychosocial impact), which were subject to review through cognitive interviews with patients and feedback from clinical experts. The 29-item PROM is now being field-tested in seven clinics in four countries.

- Clinical relevance: SCAR-Q demonstrates the most rigorous content validation process.
- Reliability: Preliminary findings show good to excellent reliability (27).

## Discussion

Currently, there are no assessment tools that convincingly demonstrate intra- and inter-rater reliability, validity and responsiveness to clinical changes. An overview of some of the more widely-utilised tools is shown in Tables 1A,B. Unfortunately, many of the tools described above fulfil neither the rigorous methodology nor health measurement principles required in other areas of medicine. The clinimetric properties and assessment criteria of these objective measures are summarised in Tables 1A,B, respectively.

**Table 1B:** Criteria of current assessment tools.

**Assessment tool**	**Colour**	**Thickness**	**Relief**	**Pliability**	**Surface area**	**Clinician's general assessment**	**Patient's general assessment**	**Co-morbidities (pain, pruritus)**	**Functional/psycho-social outcomes**
Minolta chromameter	✓								
Mexameter	✓								
Biopsy		✓							
TUPS		✓							
Silflo			✓						
Skin elasticity metre				✓					
Tonometry				✓					
Scar-tracing					✓				
Photography					✓				
Stereophotogrammy					✓				
Rating photographs	✓	✓				✓			
WES									
MSS	✓	✓							
VSS (modified)	✓	✓		✓				✓	
POSAS	✓	✓	✓	✓		✓	✓	✓	
BBSIP	✓						✓	✓	✓
BSHS								✓	✓
Bock								✓	✓
PSAQ	✓		✓		✓			✓	✓
PRISM							✓	✓	✓
SCAR-Q	✓		✓		✓			✓	✓

*TUPS, Tissue Ultrasound Palpation System; WES, Wound Evaluation Scale; MSS, Manchester Scar Scale; VSS, Vancouver Scar Scale; POSAS, Patient and Observer Scar Assessment Scale; PSAQ, Patient Scar Assessment Questionnaire; PRISM, Patient-Reported Impact of Scars Measure; BBSIP, Brisbane Burn Scar Impact Profile*.

PROMs are a beneficial addition to the armamentarium of scar assessment tools. They provide the most direct measure of patient satisfaction and quality of life, and are relatively feasible to administer. However, existing instruments still have limitations. In terms of instrument content, only PRISM and SCAR-Q have demonstrated a rigorous content design and validation process by using qualitative patient data. PRISM attempts to encompass both symptomatic and psychosocial aspects of scars, but only SCAR-Q integrates scar appearance, symptoms, and psychosocial impact.

None of the PROMs available are universally applicable across clinical contexts. For example, BBSIP is only purposed for burns patients. While SCAR-Q is the first instrument designed to be applied to all scar types, the qualitative datasets used in content development did not include burn scars. POSAS has the advantage for being widely-validated and used across different scar types—this could better facilitate the development of greater consensus on significant scar features and psychosocial aspects.

On balance, SCAR-Q is a promising tool but further studies are needed to demonstrate its utility across clinical and cross-cultural contexts. The vast majority of these tools are most useful to assess large cohorts of patients both within trials and in clinical scar services. On an individual patient level, these measures must be integrated with clinical judgement, patient priorities, social factors, and a thorough multidisciplinary assessment. Validated scoring systems therefore provide an additional window into scar and patient assessment but cannot stand alone.

## How to Use Scar Assessment Tools

**Figure 1:**
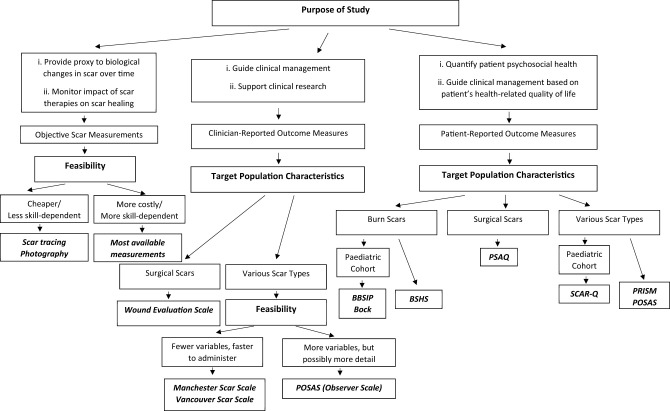
A proposed guide to how to use currently available scar assessment tools.

Despite their limitations, different tools with different clinimetric qualities can still provide useful clinical information. Tyack et al. have published a guide to choosing a burn scar rating scale for clinical or research use (28). Figure 1 provides a concise algorithm to guide the selection of an appropriate scar assessment tool.

### Purpose of the Study

Objective scar measurements could be used to monitor benefits of scar treatment, by providing proxies to biological changes occurring within tissue. This may be useful for research on scar therapies. However, the scar's physical attributes may be less relevant for studying patients' psychosocial health, which may be a focus of clinical studies. Lawrence et al. (29) examined the relationship among burn scarring, severity and visibility, and body esteem (29). The visibility of scarring was unrelated to self-satisfaction. When multiple regression analysis for predicting body esteem was performed, burn characteristics accounted for <20% of the variance. Conversely, social adjustment and depression accounted for the most variance. Hence, PROMs may provide a better measure of psychosocial health and HRQoL. In particular, POSAS can reflect changes in HRQoL over time. This can help clinicians evaluate the psychosocial health of their patients as they progress in their physical recovery.

### Target Population Characteristics

Many of the scales have yet to be assessed in other cultural and/or ethnic contexts, although POSAS has been demonstrated to be transferable to other cultural populations (16, 17). Only a few measures (BBSIP and Bock) have been validated in paediatric populations. Other tools may be less accessible to a paediatric population.

The context of the scar may also be important. Scars caused by accidents and assault usually have greater psychological impact (30). These patients can experience higher scores on the General Self-Consciousness (GSC) scale as compared to patients with sports-related facial scars (31). Thus, both objective and subjective assessments may be required for a holistic representation of patient health. Burn scars may present unique challenges in restoring appearance, function and quality of life, so it may be appropriate to employ a burn-specific assessment tool. However, it has not yet been shown if burn-specific measures like BSHS and BBSIP are superior to POSAS (which has been applied to burn scars).

### Feasibility

In low-resource settings, highly specialised, or costly tools (including spectrophotometry and stereophotogrammetry) are unfeasible. CROMs and PROMs can be easily administered within an outpatient clinic setting. For example, POSAS is reported to take <5 min to administer (28). However, PROMs are unfeasible when patients are unconscious, unable to understand or complete questionnaires.

## Conclusions

A patient with a scar does not only see measurable characteristics (pigmentation, thickness, length, etc.) but also experiences unseen discomforts (like itch and pain). There may be sequelae such as self-consciousness, loss of identity, isolation, and depression. How others evaluate the scar can also contribute to the patient's psychosocial health. Unfortunately, these factors have yet to be fully assimilated into scar assessment tools. More qualitative studies are needed to understand the patient's experience of scarring. A greater synthesis of the quantitative and qualitative is needed, so as to produce a comprehensive and holistic assessment. Tools need to be meaningfully applied in the appropriate contexts to help direct treatment, monitor scar evolution, and allow the robust evaluation of therapies. Additionally, these tools need to be rigorously tested for validity and responsiveness, so that they will provide a common platform to engage in academic and clinical discussion.

## Author Contributions

AC conducted the literature review and drafted the submission. YO and FI oversaw the editing process. All authors contributed to the paper and approved the final submission.

## Conflict of Interest

The authors declare that the research was conducted in the absence of any commercial or financial relationships that could be construed as a potential conflict of interest.
