# Supplementary material for: Scar Assessment Tools: How Do They Compare?
Source: Front Surg. 2021 Jun 23;8:643098. doi: 10.3389/fsurg.2021.643098 (PMC8260845; doi:10.3389/fsurg.2021.643098)
Supplement: Supplementary file 1 [file Data_Sheet_1.docx]

**SUPPLEMENTARY MATERIAL: OBJECTIVE SCAR MEASUREMENTS**

*Colour*

- Available tools: **Tristimulus reflectance colorimetry** and **narrow-band spectrophotometry**
- Clinical relevance: Scar colour may have significant influence on observers’ opinion of a scar (albeit not for patients) [13]. Scar vascularity is associated with formation of hypertrophic or keloid scars, giving this clinical importance [1].
- Reliability: Tristimulus reflectance colorimetry has been shown to have good inter-observer reliability and validity [33].
- Feasibility: The devices used for tristimulus reflectance colorimetry (e.g. the Minolta Chromameter CR-200 and CR-300) and narrow-band spectrophotometry (e.g. Mexameter) may be too cumbersome for use for every day practice [33].

*Thickness*

- Available tools: **Biopsy** and **Tissue Ultrasound Palpation System (TUPS)**
- Clinical relevance: Scar thickness influences both observers’ and patients’ opinion of scar appearance. TUPS has shown only moderate validity [37].
- Reliability: Both measures are reliable [37].
- Feasibility: The biopsy may be too invasive for frequent clinical use.

*Relief*

- Available tools: The **Silflo silicon polymer** can be used to create a negative replica of the skin and its topography analysed [35].
- Clinical relevance: The roughness of the scar can influence observers’ opinion [13] and could predict long-term scar quality.
- Reliability: This method has shown reliability [5], and has been used in various studies [35].
- Feasibility: The process is time-consuming and challenging for clinical use.

*Pliability*

- Available tools: **Cutometer Skin Elasticity Meter** and **Tonometry**
- Clinical relevance: This feature is included in several assessment tools, but its importance to patients and clinicians has not been established.
- Reliability: The elasticity meter has shown reasonable reliability, but the tonometry measurement can be affected by underlying bony structures [39].
- Feasibility: The elasticity meter is relatively easy and efficient to use.

*Surface Area*

- Available tools: **Scar tracing**, **Photography**, **and Stereophotogrammetry**
- Clinical relevance: Measuring surface area can help assess for scar contraction or expansion.
- Reliability: Scar tracing has been shown to be fairly reliable [38]. Photographic assessment can be made more precise using stereophotogrammetry [38].
- Feasibility: Scar tracing is simple and commonly-used, whereas the stereophotogrammetry technique is specialised, time-consuming and costly [5].

**EVALUATION OF OBJECTIVE SCAR MEASUREMENTS**

While scar measurements might be seen as an objective and quantifiable way to assess scars, none of the available tools combine clinical relevance and feasibility. As a result, these tools are not commonly utilised in clinical practice. On the other hand, Clinician-Reported Outcome Measures and Patient-Reported Outcome Measures are relatively easy to administer within an outpatient clinic setting and could directly address scar features important to patients and their clinicians.
